# Histological study of human periodontal tissue following biologically oriented preparation technique (BOPT)

**DOI:** 10.4317/jced.56290

**Published:** 2020-06-01

**Authors:** Rubén Agustín-Panadero, José-Javier Martín-de Llano, Antonio Fons-Font, Carmen Carda

**Affiliations:** 1 DDS, PhD. Adjunct Professor, Department of Oral Medicine, Faculty of Medicine and Dentistry, University of Valencia, Spain; 2 BSc, PhD. Professor, Department of Pathology and Health Research Institute of the Hospital Clínico (INCLIVA), Faculty of Medicine and Dentistry, University of Valencia, Spain; 3 DMD, PhD. Professor, Department of Oral Medicine, Faculty of Medicine and Dentistry, University of Valencia, Spain; 4 DMD, PhD. Chairman, Department of Pathology and Health Research Institute of the Hospital Clínico (INCLIVA), Faculty of Medicine and Dentistry, University of Valencia, Spain

## Abstract

**Background:**

The aim of this study was to conduct histological analysis of a human tooth resected with the periodontal insertion apparatus intact following treatment using biologically oriented preparation technique (BOPT).

**Material and Methods:**

This descriptive histological dento-periodontal study used an anterior tooth extracted with the surrounding periodontal tissues intact, following prosthetic restoration with BOPT. The sample patient was recruited from among those attending the Department of Dental Medicine at the Faculty of Medicine and Dentistry, University of Valencia (Spain). Eight serial sections of the restored tooth were processed. The relative location and histological characteristics of the cemented prosthetic crown, the dental tissues of the tooth prepared by BOPT technique, and the periodontal tissues were analyzed.

**Results:**

Structural analysis of the neoformed junctional epithelium showed that the number of layers decrease apically until there was a single row of cells perfectly adhered to the acellular cementum, and beneath the epithelium a connective tissue evidently free from inflammation. The tissues of the neoformed periodontium (gingival ligament, sulcular epithelium, junctional epithelium) presented histologic normality.

**Conclusions:**

Biologically oriented preparation technique is a reliable alternative to conventional horizontal finish lines.

** Key words:**Vertical preparation, prosthetic cementoenamel junction (PCEJ), finish line, BOPT, crown.

## Introduction

Tooth preparation technique without finish line, also known as biologically oriented preparation technique (BOPT), is a protocol in which the emergence of the tooth crown is eliminated above its cemento-enamel junction (CEJ) using diamond burs (1). This makes it possible to create a prosthetic anatomical full coverage crown that will help periodontal tissues to structure themselves and stabilize around the cervical area (2).

Research conducted to date vouches for good periodontal behavior around teeth prepared by BOPT, and for the stability of peri-coronal soft tissues (3). In BOPT, both the tooth and the gingiva are drilled, creating an axial plane by means of vertical tooth reduction. During drilling, the bur interacts with both the tooth surface and the epithelial component of the gingival insertion, carefully regulating sulcular and junctional epithelium (4). The bleeding provoked helps to produce the formation of new epithelium up to the anatomical edge of a provisional prosthesis. The formation and stabilization of this new epithelium is the key factor to ensuring a successful outcome. So, although the procedure makes use of prosthodontic components, in reality BOPT involves modification of the periodontal tissues (1-4).

The aim of this study was to conduct histological analysis of a human tooth resected with the periodontal insertion apparatus intact following treatment using BOPT.

Materials and Methods

This descriptive histological dento-periodontal study used an anterior tooth extracted with the surrounding periodontal tissues intact, following prosthetic restoration with BOPT. The University of Valencia Ethics Committee for Research Involving Human Subjects approved the study protocol (Registration number H1549016627287, 7th February 2019). The study was carried out at the Department of Pathology, Faculty of Medicine and Dentistry, University of Valencia (Spain).

The criteria for obtaining the sample were as follows: Inclusion criteria: Age >18 years; good general health; non-smoker or smoker consuming <10 cigarettes per day; patient presenting an anterior maxillary or mandibular tooth with poor prosthetic prognosis requiring extraction; patient willing to provide informed consent to take part in the study. Exclusion criteria: active periodontal disease around the tooth, unmanaged diabetes or any other systemic disease that could compromise surgery; in treatment by bisphosphonates, pregnant or lactating women.

The sample patient was recruited from among those attending the Department of Dental Medicine at the Faculty of Medicine and Dentistry, University of Valencia (Spain). The female patient chosen was aged 63 years and presented a single upper right central incisor. This tooth was chosen for its poor restorative prognosis and severe atrophy of the alveolar ridge in the edentulous area which, together with the extrusion of the peri-dental tissues produced a large discrepancy, making prosthodontic treatment difficult. The treatment plan consisted of a first phase of tooth extraction and pre-prosthetic bone remodeling surgery. It was decided that this case would provide an ideal specimen for the purposes of the present study as it was possible, through the use of therapeutic strategies, to extract the cervical part of the periodontal tissue together with the tooth.

Following Helsinki Declaration guidelines for experiments involving human subjects, we explained the surgical treatment to the patient, providing full details of the procedure, and of the need to return for later check-ups. Via an informed consent form, the patient accepted a 16 weeks delay before treatment; during this time a full coverage crown would be placed with BOPT technique in order to study the soft tissues responses after BOPT. All personal data and medical notes related to treatment were subject to complete confidentiality by the staff involved in the study, in accord with Spanish legislation.

As in any treatment involving a fixed prosthesis, the first step is to carry out intra and extraoral examination taking clinical notes, photographic records, and then to fabricate study models set in a semi-adjustable articulator. After diagnosis and treatment planning, the models set in the articulator were sent to the laboratory technician who fabricated a provisional crown in self-polymerizing acrylic resin (Sintodent; Dental Ibérica).

For dental preparation without finish line, the drilling system and adaptation procedure for placing the full coverage provisional crown were as described by Agustín (2-4), with the following sequence: Double Probing (the depth of the gingival sulcus and the distance from the gingival margin to the bone crest were measured). It is essential to establish the exact distance to the bone level to prevent drilling from damaging this structure. At this point, the location of the cemento-enamel junction must be determined as drilling must reach a point 1 mm apical of this anatomical feature. To do this, the patient was anesthetized and a millimeter calibrated periodontal probe inserted in the gingival sulcus, parallel to the tooth’s axis, measuring from the bone crest. Once the probe is placed on the bone and supported laterally against the tooth, it is gently removed in coronal direction, the tip following the anatomical features until it locates the CEJ, the point at which the anatomical crown starts. Drilling begins with a 2 mm reduction of the incisal edge and chamfering of the vestibular face of the incisal third to an angle of approximately 45º. Reduction of the axial walls is performed with a diamond cone bur, with a diameter of 1.4 mm, creating a curved chamfered finish line; reduction was of 1 mm on all the tooth’s axial walls, creating a supragingival finish line 2 mm from the margin. At this stage the gingival area was not drilled. Intrasulcular instrumentation was carried out by means of simultaneous drilling, reducing dental structure while producing rotary gingival curettage using a flame-shaped diamond bur with a granulometry of 100/200 microns and a diameter of 1.2 mm. The bur was inserted in the gingival sulcus at an oblique angle of between 10 and 15º to the tooth’s axis, so that the dental face was drilled with the internal side of the bur, while gingival tissue was drilled with the external side and the tip. When the first millimeter of anatomical emergence had been drilled, the angle of the drill was altered to avoid damaging the root, placing it parallel to the tooth axis; in this way, the anatomical crown’s convexity was eliminated above the CEJ. To complete preparation, the bur was angled slightly in occlusal/incisal direction to create the correct convergence of the tooth’s axial walls. When the tooth and the internal wall of the gingiva are drilled simultaneously, creating a plane parallel to the tooth’s axis, this also provokes a carefully managed deepithelialization of sulcular free gingiva and junctional epithelium (gingitage technique). This process seeks to provoke blood coagulate in the apical part of the preparation, stabilized by the convex cervical anatomy of the provisional crown. The aim is to facilitate the arrival of immature connective tissue cells and epithelial stem cells, stimulating cell differentiation in this favorable environment, which forms new periodontal tissue around the prosthetic emergence morphology. The edge of the provisional crown with its anatomical morphology will be located in the gingival sulcus at a depth of 0.5-1 mm, respecting biological space, so that the intrasulcular area of the provisional crown supports the gingival margin circumferentially. The healing process determines reinsertion and gingival tissue thickening adapted to this new emergence profile. The provisional prosthesis must not be removed for four weeks to avoid disturbing gingival healing. Later on, while complete maturation of the soft tissues develops (8-12 weeks), the provisional crown can be modified to achieve correct modeling of the gingival margin.

**Figure 1:**
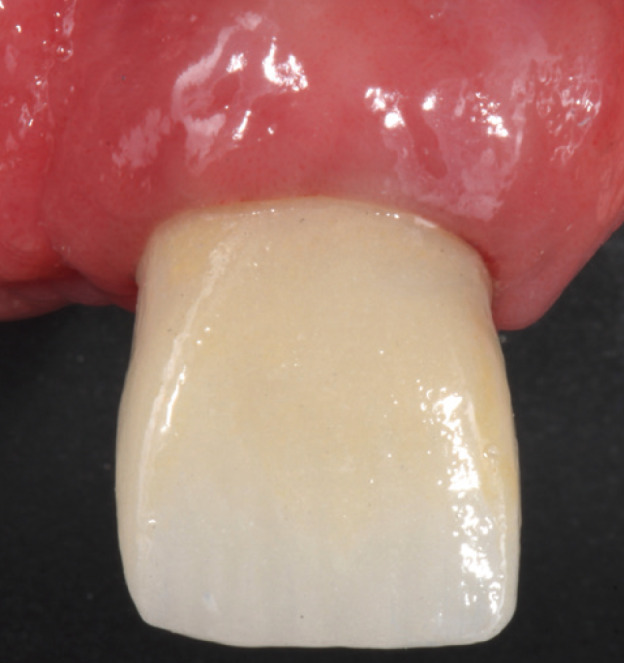
Clinical image of the definitive restoration, 1 month after cementing.

**Figure 2:**
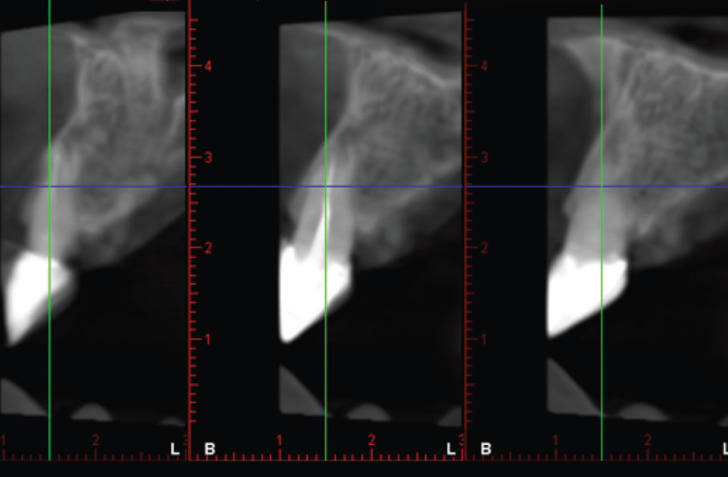
Cone Beam Computed Tomography (CBCT) of restored tooth showing the presence of cortical bone on the vestibular and palatine aspects.

After this stage, a definitive complete coverage crown was fabricated from lithium disilicate (IPS emax. Ceram; Ivoclar Vivadent) using ceramic injection (Fig. 1). It was cemented in place under conditions of absolute isolation using a dual-polymerization resin cement (Relyx Unicem; 3MESPE) (Fig. 2).

**Figure 3:**
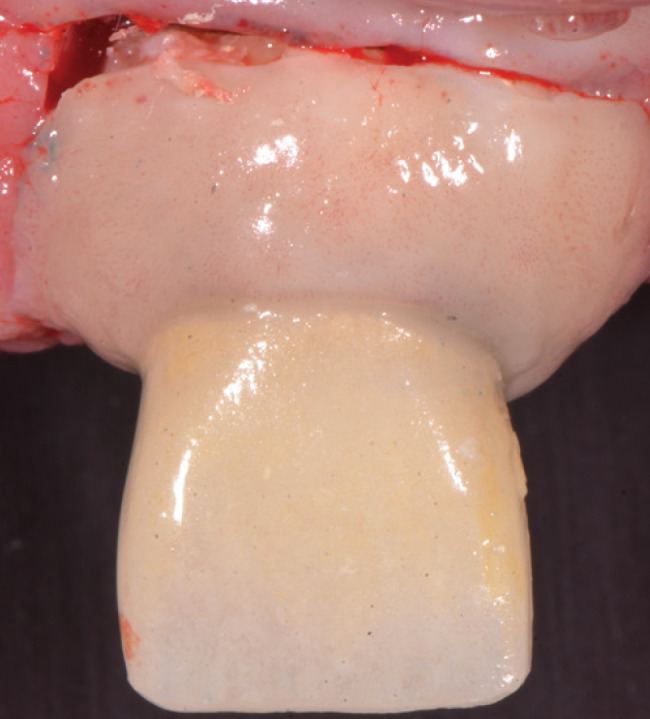
Clinical image of dento-periodontal resection.

A month after placement of the definitive restoration, the tooth and adjacent periodontal tissues (insertion and protection periodontium) were resected. To obtain the specimen, a cut was made with a piezoelectric instrument at low speed (*Pi*ezosurgery; Mectron Dental) and with saline irrigation applying a force vector perpendicular to the root axis 3 mm apical of the gingival margin. A horizontal cut was made resecting the gingiva, vestibular bone crest, cervical area of the root, cortical bone and palatine gingiva (Fig. 3).

After surgery, the sample was immersed in 10 % buffered formalin solution, fixed at room temperature for 5 days, and dehydrated by sequential solvent exchange in 70 % ethanol, 99 % ethanol and xylol (twice), for 24 h each. The sample was sequentially embedded at room temperature in 40 mL of the following solutions: methyl methacrylate (MM) for 15 days, then MM containing benzoyl peroxide (1 g/100 mL) for 3 days, and finally in MM containing poly (methyl methacrylate) (average Mw ~996,000; 5 g/100 mL) and benzoyl peroxide. Polymerization at room temperature took several days. Using a diamond wire saw (WELL Precision Vertical Diamond Wire Saw; Agar Scientific), the sample was sectioned transversally in vestibular-palatine direction in 800 μm slices. Sample slices were glued on poly methyl methacrylate slides, wet ground to a thickness of 80 μm and polished using a LaboPol-21 system (Struers) and SiC foils and further polished with diamond paste of decreasing grain sizes. The slides were sequentially stained with Stevenel’s blue and van Gieson’s picro-fuchsin following the procedure described by Maniatopoulos *et al.* (5).Digital images were recorded with a bright field Leica DM4000 B microscope and a DFC420 digital camera.

## Results

**Figure 4:**
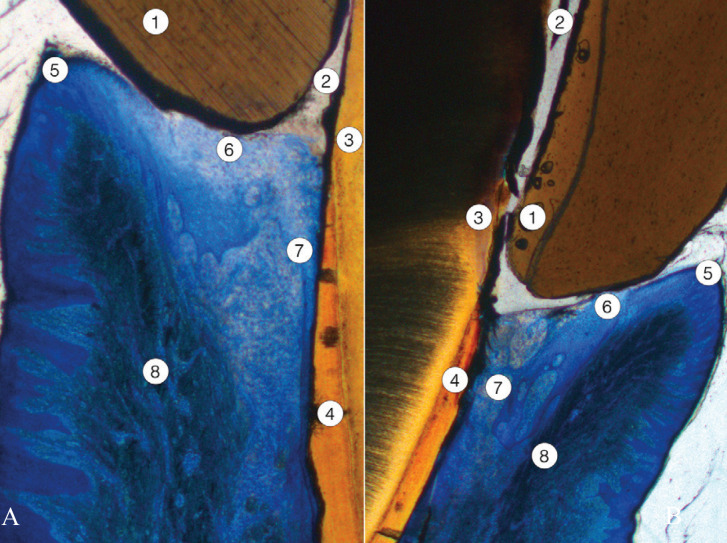
Low magnification image of two sections (A and B) of the ceramic crown cemented on the BOPT-shaped tooth. Normal periodontium has formed 12 weeks after restoration. 1: ceramic crown; 2: cementing material; 3: normal dentin, shaped in the preparation process; 4: acellular cementum shaped in the preparation process; 5: free gingival epithelium (keratinized stratified squamous epithelium); 6: sulcular epithelium; 7: junctional epithelium, attached to the cementum surface; 8: gingival ligament.

Eight serial sections of the restored tooth were processed. The relative location and histological characteristics of the cemented prosthetic crown, the dental tissues of the tooth prepared by BOPT technique, and the periodontal tissues were analyzed. In particular, newly formed tissues following restoration – sulcular and junctional epithelia and gingival ligament – were studied in detail (Fig. 4).

**Figure 5:**
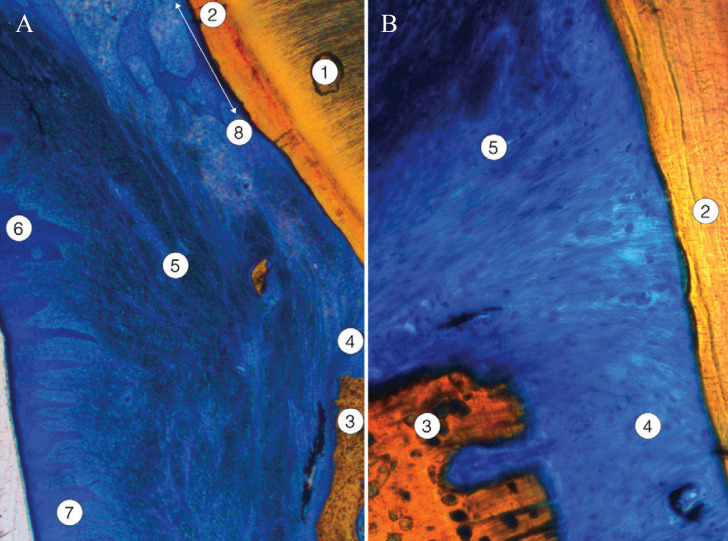
One section of the BOPT-shaped tooth showing free and attached gingiva, newly formed junctional epithelium, and periodontal ligament. A, The junctional epithelium shown was short and was not present in the zone close to the alveolar bone crest. B, Detail of the transition zone between the gingival ligament and the periodontal ligament attached to the periodontal compact and the acellular cementum. 1: dentin; 2: acellular cementum, unshaped; 3: alveolar bone crest; 4: periodontal ligament; 5: gingival ligament; 6: free gingiva; 7: attached gingiva; 8: deeper portion of junctional epithelium (two-headed arrow).

**Figure 6:**
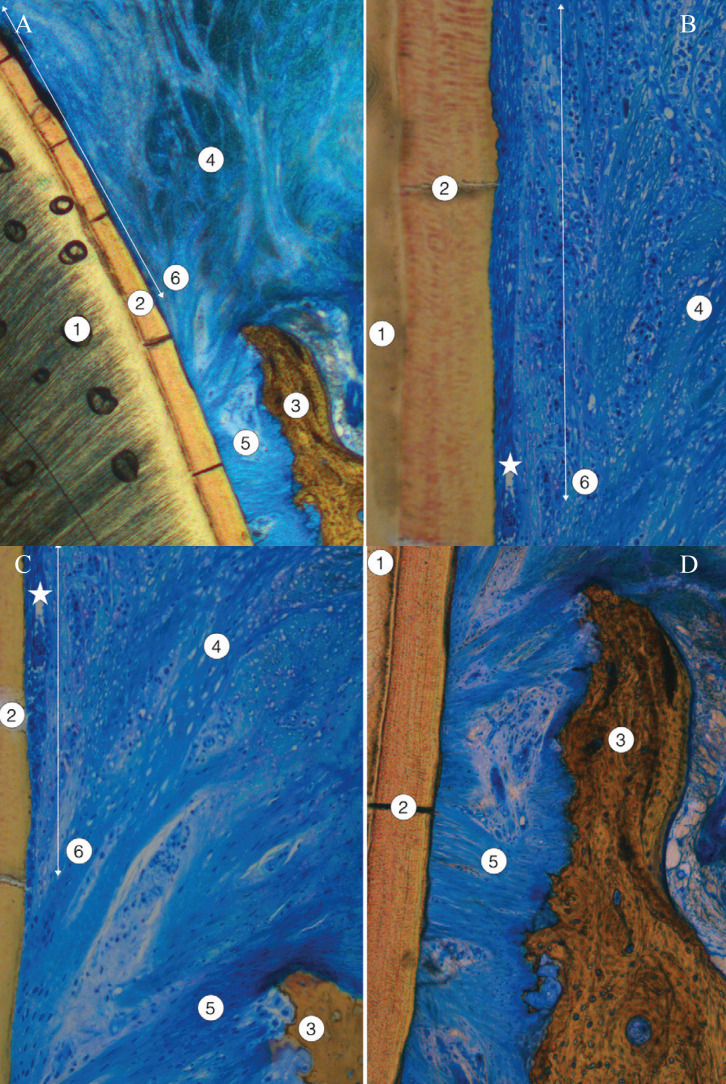
Another section of the tooth shaped by BOPT showing free and attached gingiva, newly formed junctional epithelium, and periodontal ligament. A, The junctional epithelium observed was long. B & C, Consecutive images (B coronal and C apical, asterisk shows overlapping area) of the transition zone between the gingival ligament and the periodontal ligament attached to the periodontal compact and the acellular cementum. The junctional epithelium was longer and became thinner as it approached the zone corresponding to periodontal ligament insertion. D, Periodontal ligament and crest of the alveolar bone. 1: dentin; 2: acellular cementum; 3: crest of the alveolar bone, periodontal compact; 4: gingival ligament; 5: periodontal ligament; 6: deeper portion of junctional epithelium (two-headed arrow).

As a result of the BOPT process, the absence of enamel and the presence of normal coronal dentin without histological alterations were evident. The partial removal of acellular cementum by BOPT technique did not modify the structure of the underlying root dentin (Figs. 5,6).

Towards the apical zone, the structure of the unaltered cementum and the compact bone of the alveolar crest were normal. The periodontal ligament embedded in cementum and periodontal bone showed a normal amount and spatial organization of collagen bundles, cell density and vascularization, with no signs of inflammation.

Attached gingiva was covered by a keratinized stratified squamous epithelium lining a normal connective tissue without any signs of inflammation. The free gingiva, gingival margin and sulcular epithelium regenerated following BOPT also presented a normal histological structure. The connective tissue beneath showed a slightly increased number of defensive cells but without apparent signs of inflammation. The organization and spatial distribution of the collagen bundles corresponding to the dento-gingival and dento-periosteal fascicles were normal.

Lastly, the newly formed junctional epithelium attached to the new dentin-cementum surface created after BOPT reached from the bottom of the gingival sulcus to a variable height depending on the serial section studied. In some sections a short and wider junctional epithelium was observed, far from the alveolar crest. In other sections the epithelial attachment was much longer, lining the dental surface close to the alveolar bone crest. This was newly formed epithelium, and the number of layers decreased apically, ending with a single row of cells firmly attached to the non-carved acellular cementum (Fig. 6).

## Discussion

When teeth are restored with fixed prostheses, the location of the gingival margin has always been a subject of debate within the field of dentistry (6-8). Some authors argue in favor of placing the prosthetic margin away from the epithelial insertion (juxta- or supragingival) to eliminate any factor that could provoke gingival inflammation (6,7). But others have not found statistically significant differences arising from the location of the prosthesis’ gingival margin (8). In some cases, the dentist has no alternative but to situate the gingival margin inside the sulcus, for example, in cases of oblique subgingival tooth fracture, cases of root caries, when the dental stump presents a dark color, when there is sensitivity, cervical abrasion, or insufficient retention due to a short tooth (1).

The type of dental finish line has also been widely studied in prosthodontic research during the last 35 years or more. Pardo (9), in 1982, described types of dental preparation in relation to the design of complete coverage fixed prostheses. The horizontal finish lines reported included the flat chamfer, the straight shoulder, the sloping shoulder, the vertical shoulder, the beveled shoulder, the 120º shoulder, etc. Vertical shoulders included the knife-edge finish line, which is free-sliding and conventionally used for cast metal prosthetic finish lines. More recently, Loi (1) described the biologically oriented preparation technique (BOPT), based on vertical preparation, which consists of carving the tooth to create a vertical plane between the dental anatomical crown and the root area. The tooth reduced by BOPT does not have a finish line (nor is it a knife-edge finish line), as the finish line is defined by the edge of the prosthetic restoration and is characterized by contouring the crown in the cervical area in relation to periodontal parameters. Although BOPT is a fairly recent development, several short-medium term prospective studies have reported good perio-prosthetic clinical behavior, observing stability of the gingival margin, and increased gingival thickness in the area of prosthetic emergence, which is the main factor of concern to restorative dentists (3,5).

Following the destructuring of periodontal tissue caused by dental preparation and shaping, designed to simulate the anatomy of the natural tooth, healing and physiological maturation of the periodontal tissues takes place in such a way that these adapt to the contours of the prosthesis’ cervical emergence.

In the present study, structural analysis of the neoformed junctional epithelium showed that the number of layers decrease apically until there was a single row of cells perfectly adhered to the acellular cementum, and beneath the epithelium a connective tissue evidently free from inflammation. In particular, the tissues of the neoformed periodontium (gingival ligament, sulcular epithelium, junctional epithelium) presented histologic normality.

## Conclusions

Biologically oriented preparation technique is a reliable alternative to conventional horizontal finish lines. After BOPT, in which gingival tissues are modeled in response to the shape of the prosthetic emergence, periodontal protection tissues, especially neoformed insertion tissue, are structurally equivalent to normal periodontal tissue, ensuring a healthy environment free from inflammation.
